# OPAA template-directed synthesis and optical properties of metal nanocrystals

**DOI:** 10.1186/1556-276X-8-328

**Published:** 2013-07-18

**Authors:** Xiu-chun Yang, Jun-wei Hou, Yan Liu, Miao-miao Cui, Wei Lu

**Affiliations:** 1 Department of Materials Science and Engineering, Tongji University, Shanghai, 201804, China; 2 Research Institute of Experiment and Detection, PetroChina Xinjiang Oilfield Company, Karamay, Xinjiang, 834000, China

**Keywords:** Nanostructures, Chemical synthesis, Electron microscopy, Optical properties

## Abstract

Ag and Cu nanocrystals (NCs) were assembled into ordered porous anodic alumina (OPAA) by a single-potential-step chronoamperometry technique. The composition, morphology, microstructure, and optical property were analyzed by X-ray diffraction, field-emission scanning electron microscopy, transmission electron microscopy, selected area electron diffraction, and optical absorption spectroscopy. The results indicate that metallic NCs/OPAA composite possesses a significant surface plasmon resonance absorption. For continuous electrodeposition, metallic nanowires are smooth and uniform with face-centered cubic (fcc) single-crystalline structure; however, for interval electrodeposition, the nanowires are bamboo-like or pearl-chain-like with fcc polycrystalline structure. The length of the nanoparticle nanowires or the single-crystalline nanowires can be controlled well by adjusting the experimental cycle times or the continuous depositing time. The transverse dipole resonance of metallic NCs enhances and displays a blue shift with increasing electrodeposition time or experimental cycle times, which is consistent with Zong's results but contradictory to Duan's results. The formation mechanisms of the nanoparticle nanowires and the single-crystalline nanowires were discussed in detail.

## Background

The optical properties derived from nanostructured metallo-dielectric composites have attracted worldwide attention both from experimental and theoretical aspects [1-3]. The absorption spectrum of metallic nanoparticles could be attributed to surface plasmon resonance (SPR), i.e., collective oscillations of conduction electrons driven by the incident light field. The SPR frequency depends strongly on the metal composition, structure (solid, hollow, and core shell), size and shape, the dielectric properties of the surrounding medium, and inter-nanoparticle coupling interactions [4-11]. The fascinating optical properties of metal NCs find important applications in a number of technological areas including biological imaging, sensing and detection [12-20], photothermal imaging and therapy [21,22], surface-enhanced Raman scattering [23-27], plasmon-enhanced fluorescence [28-30], integrated nanophotonic devices [31-33], photovoltaic devices [34], data storage [35,36], and nonlinear optics [37,38].

To identify whether a resonance originates from a longitudinal mode or a transverse mode, well-aligned metal nanowires represent an ideal configuration. For examples, Zong et al. [39-41] reported that a dual peak appeared when the incident light was perpendicular to the surface of the composite film of Ag nanowire arrays in anodic aluminum oxide (AAO) template. The two peaks were ascribed to the transverse dipole resonance (longer wavelength) and the transverse quadrupole resonance (shorter wavelength), respectively. The quadrupole resonance peak displayed a distinct red shifting from 350 to 365 nm and became the strong peak when the diameter reached 40 nm. Duan et al. [42] also reported that a dual peak appeared when the incident light was perpendicular to the surface of the composite film of Cu nanowire arrays in ion-track templates. The dual peak with a shorter wavelength was attributed to interband transition of Cu bulk metal, and the dual peak with a longer wavelength was ascribed to transverse dipolar peak, which displayed red a shift with increasing nanowire length. This result is obviously different from the blue shift reported by Zong et al. In order to clarify the difference, a new procedure to electrochemically fill ordered porous anodic alumina (OPAA) was developed where porous alumina remained on the aluminum substrate and the barrier layer was very thin by using a step-by-step voltage decrement process [43]. The thinning leads to a considerable decrease in the potential barrier for the electrons to tunnel through the barrier layer, when the metal is deposited at the pore tips. Ag and Cu nanocrystals (NCs) were successfully assembled into the ordered OPAA by a single-potential-step chronoamperometry technique, and the influences of preparation processes on the morphology, structure, and optical property of metallic NCs were deeply investigated.

## Methods

A highly ordered OPAA template with uniform pore diameters of about 60 nm and smooth pore channels perpendicular to the membrane surface was fabricated by a two-step anodization process plus a step-by-step voltage decrement method as described previously [43,44]. The high purity alumina foil (99.999%) with size of 2 cm × 2 cm × 0.5 mm was firstly annealed at 500°C for 5 h and ultrasonic cleaned for 3 min in acetone, ethanol, and deionized water, respectively. The native oxide layer was removed in 2 mol/L NaOH solution at 60°C for 2 min. Then, the aluminum foil was anodized in 0.3 mol/L oxalic acid aqueous solution under constant voltage (40 V) and constant temperature (5°C). After anodization for 4 h, the formed alumina was removed by a mixture solution of phosphoric and chromic acids. Afterward, the foil was anodized for 5 h again at the same condition as the first anodization. In order to promote thinning of the barrier, the anodic voltage was gradually lowered to 30 V by 2 V/min and then to 5 V by 1 V/min after the second anodic process. The anodization at 5 V continued for 10 min to allow the equilibration of the barrier layer at the pore bottom. Finally, the template was obtained by a subsequent etching treatment in 5 wt.% phosphoric acid (35°C) for 30 min. Electrodeposition was performed on LK98II electrochemical system (Lanlike, Tianjin, China) using the single-potential-step chronoamperometry technique. In the electrodeposition cell, the OPAA template with Al substrate, Pt plate, and saturated calomel electrode were used as the working electrode, the counter electrode, and the reference electrode, respectively. Samples Ag1 and Ag2 were electrochemically deposited in a mixture of 0.05 mol/L AgNO_3_ and 0.05 mol/L H_3_BO_3_ aqueous solutions at −6.5 V for 50 and 100 s, respectively. Samples Ag3, Ag4, and Ag5 were electrochemically deposited in a mixture of 0.01 mol/L AgNO_3_ and 0.01 mol/L H_3_BO_3_ aqueous solutions at a depositing potential of −6.5 V with deposition time of 2 s and interval time of 5 s. Experimental cycle times of 20, 50, and 100 were used for samples Ag3, Ag4, and Ag5, respectively. Sample Cu1 was electrochemically deposited in a mixture of 0.2 mol/L CuSO_4_ and 0.01 mol/L H_3_BO_3_ aqueous solutions at −6.0 V for 400 s. Samples Cu2, Cu3, and Cu4 were electrochemically deposited in a mixture of 0.01 mol/L Cu(NO_3_)_2_ and 0.1 mol/L H_3_BO_3_ aqueous solution at a depositing potential of −8.5 V with deposition time of 1 s and interval time of 5 s. Experimental cycle times of 150, 200, and 300 were used for samples Cu2, Cu3, and Cu4, respectively. Here, H_3_BO_3_ was used as buffer reagent. After deposition, the samples were rinsed with deionized water, and then, the Al substrate was removed by 10 wt.% CuCl_2_ aqueous solutions.

Hitachi (Chiyoda-ku, Japan) 3310 UV–vis spectrophotometer was used to measure optical absorption of these samples using an unpolarized light beam at normal incidence to the sample plane. Quanta 200 FEG scanning electron microscope (FESEM) (FEI, Hillsboro, OR, USA) with an energy-dispersive X-ray spectroscope (EDS) was used to characterize the morphology and elemental composition. H-800 transmission electron microscope (TEM) (Hitachi Ltd., Chiyoda-ku, Japan) was used to analyze the morphology and microstructure of these samples. TEM samples were prepared by immersing a small piece of Ag/OPAA or Cu/OPAA film in 2 mol/L NaOH solution for about 5 h (60°C) in order to dissolve the OPAA template. Ag NCs or Cu NCs were afterward separated out of the solution by centrifugal effects. Finally, the deposit was ultrasonically dispersed in 3 to 5 mL ethanol, and a drop of the suspended solution was placed on a Cu grid with carbon membrane for TEM observation.

## Results and discussion

### Synthesis of Ag NCs

**Figure 1:**
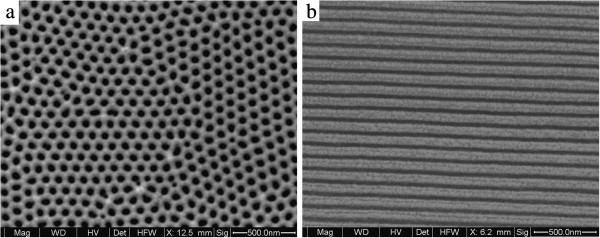
**SEM images of the OPAA template. (a)** Top view, **(b)** cross-sectional view.

Figure 1 gives SEM images of the ordered OPAA template. Figure 1a shows that the honeycomb-like template is highly ordered with circular holes and hexagonal structure cell. The pore diameter and pore density are approximately 60 nm and 1 × l0^10^ cm^−2^, respectively. Figure 1b indicates that the pore channels are smooth and parallel to each other.

**Figure 2:**
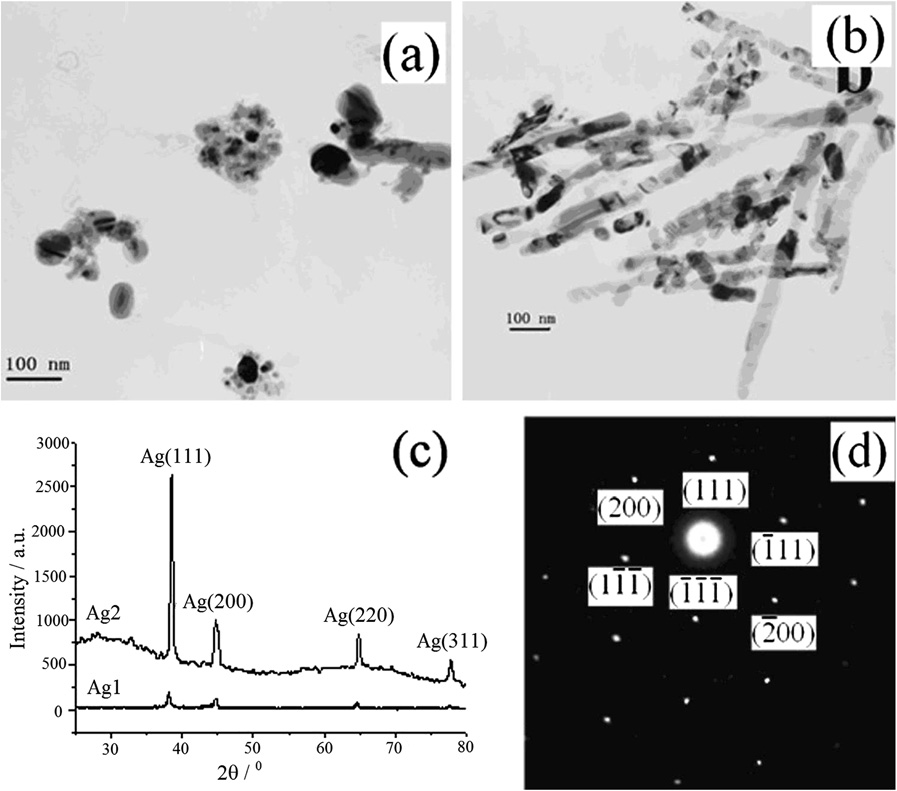
TEM images of samples Ag1 (a) and Ag2 (b); XRD pattern (c) and SAED diagram (d) of sample Ag2.

Figure 2 gives TEM images and X-ray diffraction (XRD) patterns of samples Ag1 and Ag2.

Figure 2a indicates that sample Ag1 is mainly composed of nanoparticles with a size range of 20 to 70 nm, and a few nanorods exist in the sample. Figure 2b indicates that sample Ag2 is mainly consisted of nanowires with diameters of 50 to 70 nm and an average length of 500 nm. Four peaks can be observed in the XRD patterns, as shown in Figure 2c, which correspond to (111), (200), (220), and (311) planes of face-centered cubic (fcc) silver (PDF no. 04–0783), respectively. The diffraction peak intensities are higher for sample Ag2 than sample Ag1 because sample Ag2 has a longer deposition time than sample Ag1. For sample Ag2, the (111) diffraction peak intensity is relatively higher while other peak intensities are very lower to the standard diffraction pattern of fcc Ag bulk, indicating that Ag nanocrystals were electrodeposited into the pores and grew along [111] direction as preferred orientation. As described in broken bond theory [45], fcc metals have an anisotropic surface free energy and hold a regressive sequence of (110), (100), and (111) facets. Therefore, the fcc metals such as gold, silver, copper, palladium, and nickel naturally prefer to grow with a [111] orientation [46,47], which are different from the reference's report that the fcc metals have a preferred growth orientation of [110] under direct current deposition conditions because (110) surface energy is lowest when the aspect ratio is larger than 1 [48]. Figure 2d gives the selected area electron diffraction (SAED) pattern of a nanowire in sample Ag2, indicating that the Ag nanowire possesses a single-crystalline fcc structure.

**Figure 3:**
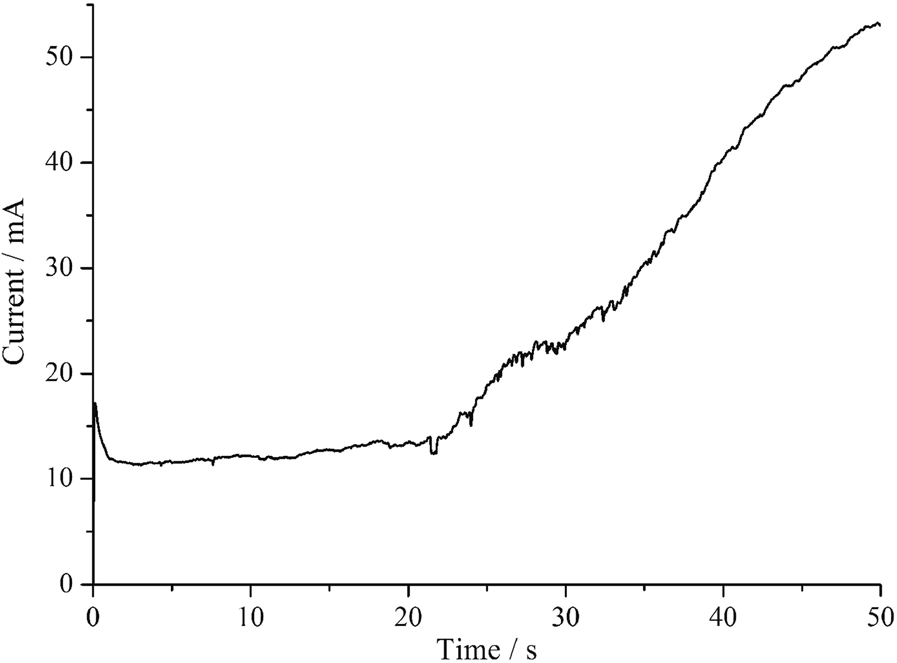
Current-time curve of sample Ag1.

In order to follow the deposition process, the current was recorded as a function of time as shown in Figure 3.

When a potential is applied, the current is large at *t* = 0 due to the charge of the electrical double layer and reduction of Ag^+^ at the cathode surface. The reduction of Ag^+^ ions at the cathode surface creates a concentration gradient that causes a flux of ions toward the cathode. In this process, the decrease of current indicates the formation of the diffusion layer. The current remains nearly constant and is very low because Ag^+^ ions diffuse slowly through the branched channel of OPAA template near the barrier layer. The current increases when some Ag nanowires grow into the main pore channel of OPAA template because of larger pore diameter and faster Ag^+^ ion diffusion in the main pore channel than in the branched channel.

**Figure 4:**
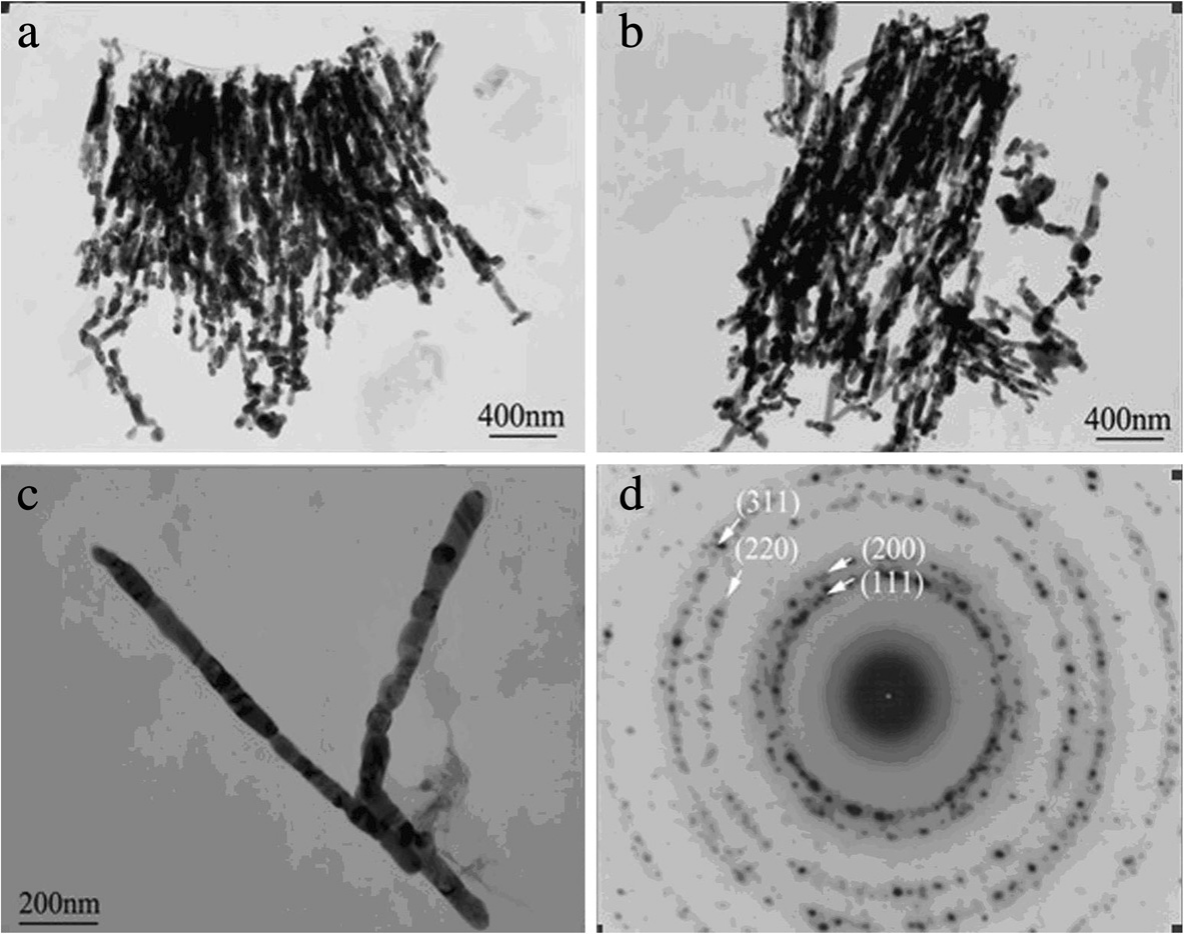
TEM images of samples Ag3 (a, c) and Ag4 (b), and SAED diagram (d) of sample Ag3.

Figure 4 gives TEM images of samples Ag3 and Ag4.

Figure 4a, b shows that the nanowires in samples Ag3 and Ag4 have nearly the same average diameter of about 70 nm and different lengths of 1 to 1.5 μm and 1.5 to 1.8 μm, respectively. The nanowire is longer in sample Ag4 due to the longer electrodeposition time. Figure 4c indicates that the nanowires have bamboo-like or pearl-chain-like structure; SAED pattern in Figure 4d indicates that the nanowires are polycrystalline with fcc structure.

**Figure 5:**
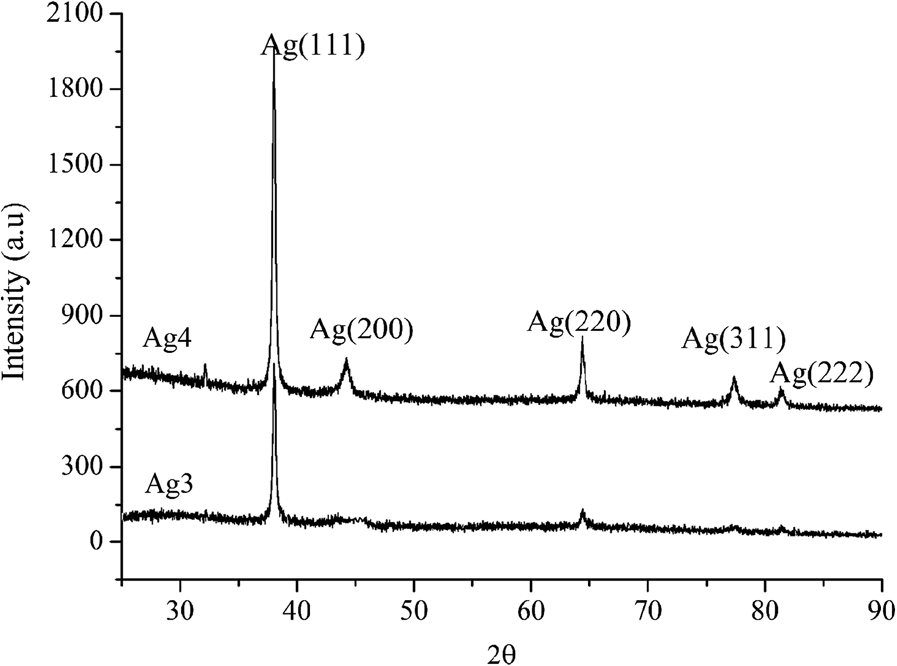
XRD patterns of samples Ag3 and Ag4.

Figure 5 gives XRD patterns of samples Ag3 and Ag4.

The XRD patterns indicate that samples Ag3 and Ag4 are composed of face-centered cubic Ag NCs, longer electrodeposition time favors the growth of Ag NCs. The calculated grain sizes are 32 nm for sample Ag3 and 29 nm for sample Ag4 based on the Scherrer's formula from (111) diffraction peaks.

**Figure 6:**
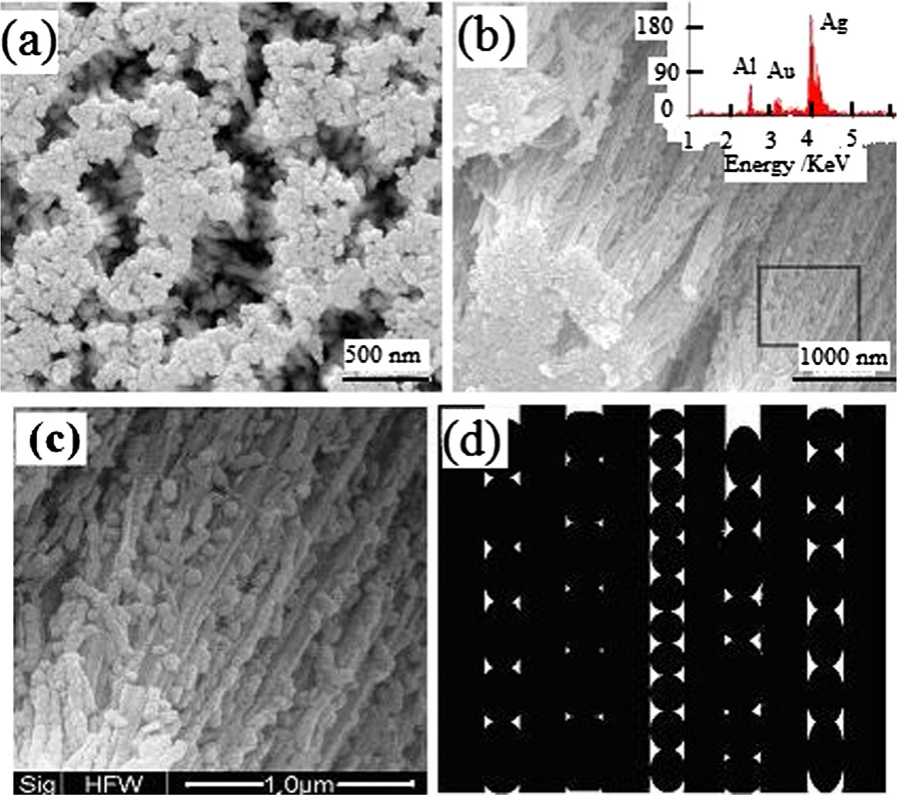
**FESEM images of sample Ag5. (a)** Top view; **(b)** cross-sectional image with an inserted EDS spectrum from the marked rectangular area; **(c)** local magnified image of **(b)**; **(d)** schematic diagram for the formation of Ag nanoparticle nanowires.

Figure 6 gives FESEM images and the corresponding EDS spectrum of sample Ag5.

Figure 6 indicates that the pores of OPAA template are highly filled by Ag nanoparticle nanowires. The Ag nanoparticles are nearly spherical, and their size distribution lies in the range of 45 to 75 nm. The Ag nanoparticle nanowires clustered together after the OPAA template was dissolved in 1 mol/L NaOH solution for 1 h. The cluster effect originates from the relatively high surface free energy of the Ag nanoparticle nanowires. The nanowires in samples Ag1 and Ag2 prepared by continuous electrodeposition are single-crystalline with smooth surface and nearly uniform diameters; however, the nanowires in samples Ag3, Ag4, and Ag5 prepared by interval electrodeposition are polycrystalline with bamboo-like or pearl-chain-like structure. For the continuous electrodeposition, Ag^+^ ions at the electrode surface are reduced into neutral Ag atoms, which nucleate and grow subsequently. This brings on a significant decrease of Ag^+^ concentration at the electrode surface because the electrophoresis diffusion of Ag^+^ ions in electrolyte is slow through the nanopore channel to the electrode. After electro-reducing, neutral Ag atoms deposit on the initial nanocrystals by epitaxial growth because the concentration of neutral Ag atoms is too low to heteronucleate on the initial nanoparticles. The epitaxial growth ensures the single-crystalline feature of Ag nanowire [46]. For the interval electrodeposition, Ag^+^ ions have enough time to diffuse to the electrode surface, after next electrodeposition, enough Ag^+^ ions are reduced into neutral Ag atoms, which can heteronucleate on the previous nanoparticles and grow into bamboo-like or pearl-chain-like nanowires, as shown in Figure 6d. The structure and morphology of nanowires depend on the preparation parameters such as the electrolyte concentration, the electrodeposition time and the interval time, the electropotential, the pore diameter, and channel morphology of the template [46,47].

### Synthesis of Cu NCs

**Figure 7:**
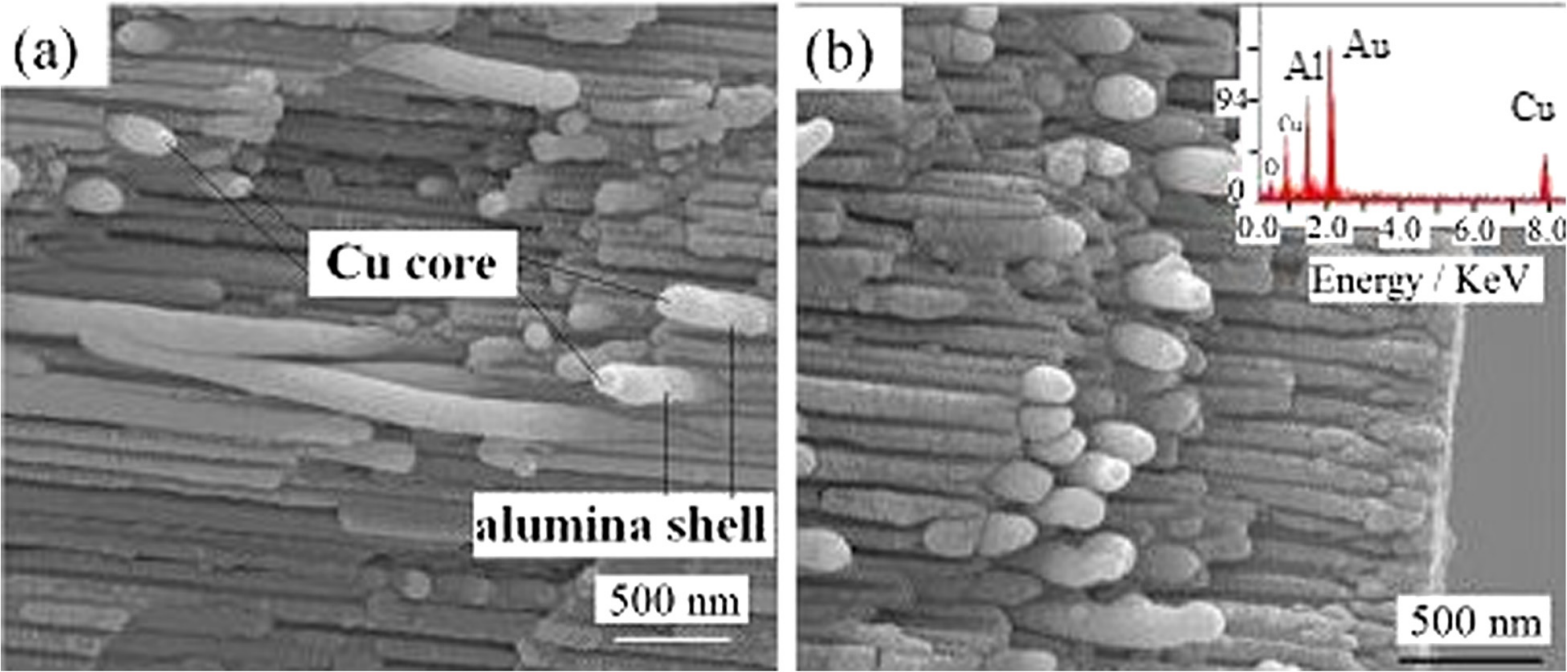
**FESEM images of sample Cu1.** (a) middle part of cross-section, (**b**) the end of cross-section.

Figure 7 gives the FESEM images of sample Cu1.

Figure 7 indicates that most nanochannels were filled by Cu nanowires with a diameter of 120 nm. The diameter is larger than the pore diameter of OPAA template because the nanowire is composed of Cu core and Al_2_O_3_ shell where the core is from Cu nanowire and the shell is from the pore wall of the OPAA template.

**Figure 8:**
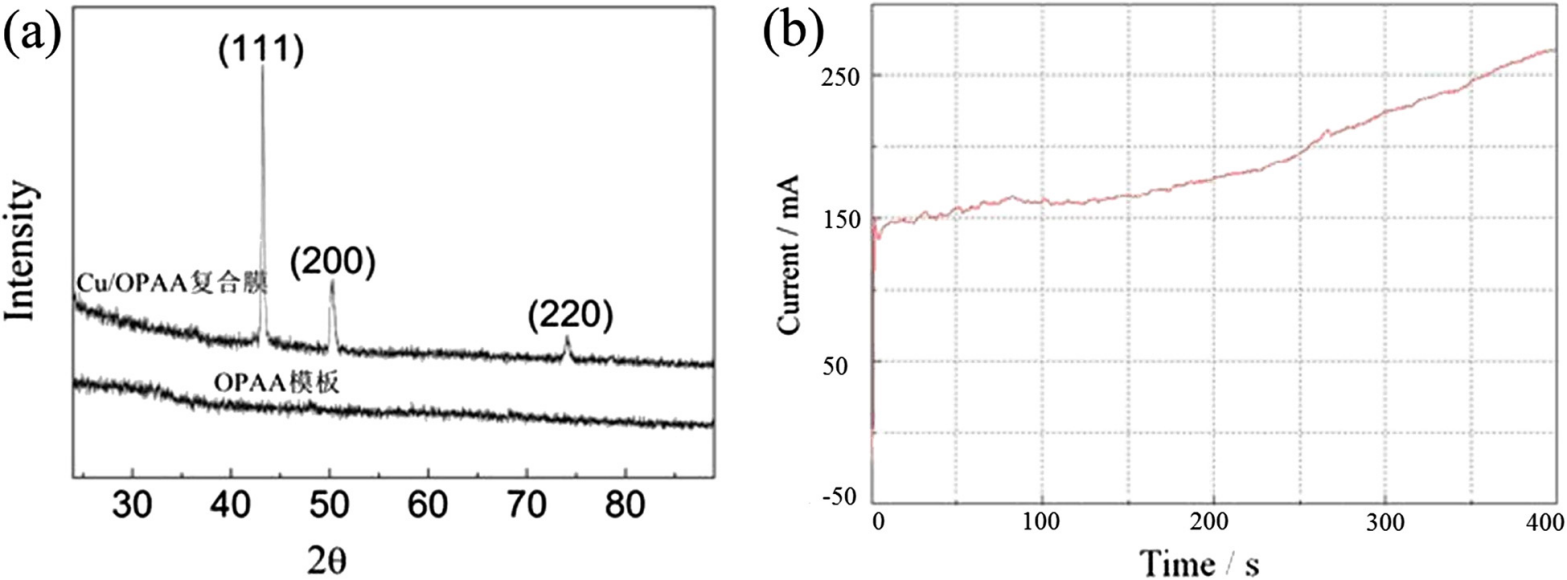
XRD pattern (a) and the current-time curve (b) of sample Cu1.

Figure 8 gives the XRD pattern and the current-time curve of sample Cu1

There diffraction peaks in Figure 8a can be indexed as (111), (200), and (220) diffraction planes of fcc Cu, respectively, which further demonstrates that sample Cu1 is composed of metallic Cu. The current rises abruptly at time zero to charge the double layer, subsequently, the current rises slowly with a little variation because Cu^2+^ ions diffuse slowly through the branched channel of OPAA template near the barrier layer. The current further increases with a higher rate after 100 s because some nanowires in branched channels grow into main pore channels of the template where Cu^2+^ ions have a higher diffusion rate.

**Figure 9:**
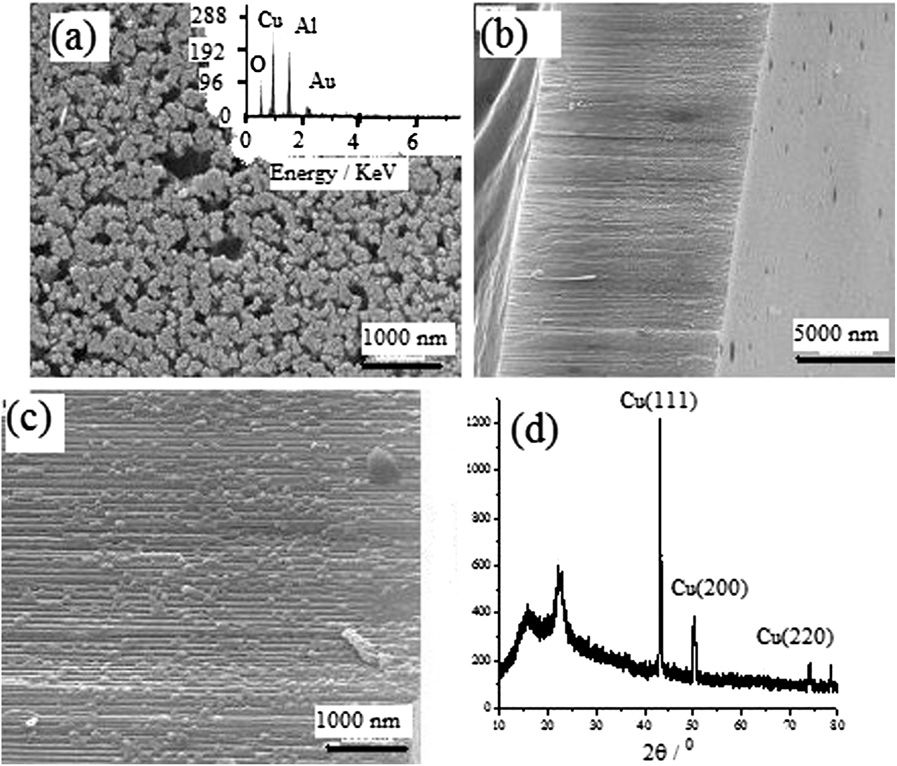
**FESEM images and XRD pattern of sample Cu4. (a)** Top view with EDS spectrum, **(b)** cross-sectional view with a low magnification, **(c)** local magnified image, **(d)** XRD pattern.

Figure 9 gives the FESEM images and XRD pattern of sample Cu4.

Figure 9a indicates that nearly all pores of the template were filled by Cu nanowires. The cross-sectional images, as shown in Figure 9b, c, indicate that the template has a thickness of 11 μm, and only 5.5-μm pore channels near the barrier layer were filled by Cu nanoparticles with long-axis diameters of 40 to 105 nm, which formed Cu nanoparticle nanowires in the pore channel. Figure 9d further demonstrates that the nanoparticle nanowires are composed of fcc Cu metal with a calculated grain size of 33 nm based on Scherrer's formula. Similar to Ag nanowires, Cu nanowires prepared by continuous electrodeposition are single-crystalline with smooth surface and nearly uniform diameter, and Cu nanowires prepared by interval electrodeposition are polycrystalline with bamboo-like or pearl-chain-like structure.

### Optical properties of metallic NCs/OPAA

**Figure 10:**
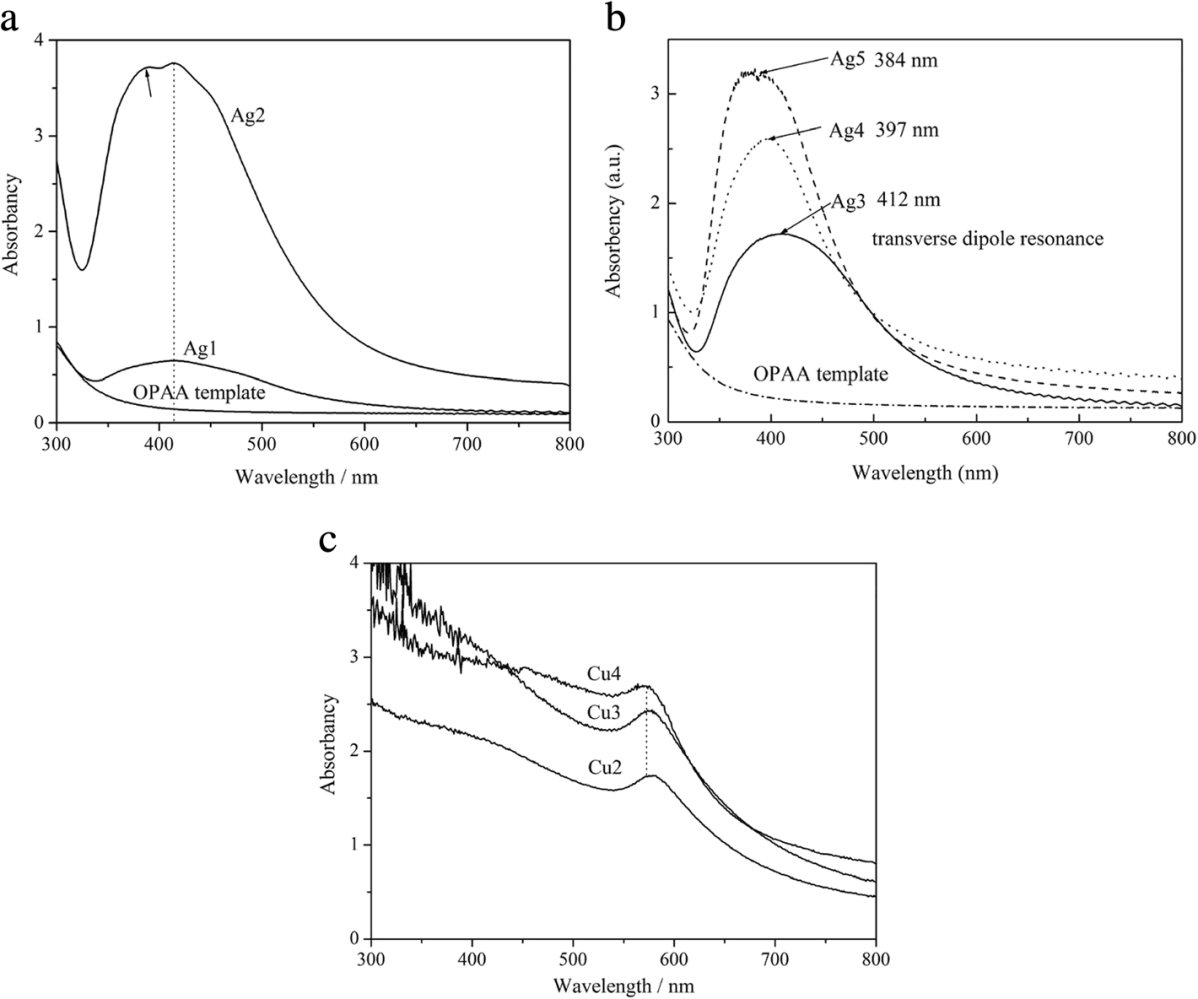
Optical absorption spectra (a) samples Ag1 and Ag2; (b) Ag3, Ag4, and Ag5; (c) Cu2, Cu3, and Cu4.

Figure 10 gives optical absorption spectra of samples Ag1, Ag2, Ag3, Ag4, and Ag5, and samples Cu2, Cu3, and Cu4.

Figure 10a indicates that the OPAA template has a very weak absorption at the wavelength longer than 350 nm, indicating that it can be an excellent matrix for fabrication of optical devices. Sample Ag2 has a dual peak at 414 and 386 nm, which is similar to Zong's results [39-41]. As well known, the longitudinal resonance cannot be excited when the unpolarized light beam is parallel to the major axis of Ag NCs. Therefore, the peak at 414 nm can be denoted as the transverse dipole resonance of Ag NCs and the peak at 386 nm can be denoted as the quadrupole resonance of Ag NCs. Zong et al. reported that the quadrupole resonance peak displays a distinct red shifting to 365 nm when the diameter reaches 40 nm. Ag nanowires in sample Ag2 have diameters of 50 to 70 nm, larger than 40 nm; therefore, its quadrupole resonance peak shifts to 386 nm. The much stronger absorption in sample Ag2 than in sample Ag1 indicates that the electrodeposition rate of Ag NCs became faster after 50 s. This is consistent with the above-mentioned results in Figures 1 and 2. Figure 9b indicates that the transverse dipole resonance of Ag NCs enhances and has a blue shift with increasing electrodeposition time, which is consistent with Gan's theory [49]. Figure 10c indicates that sample Cu2 has an absorption peak at 579 nm, which can be denoted as the transverse dipole resonance of Cu NCs. The transverse dipole resonance of Cu NCs enhances and has a little blue shift with increasing electrodeposition time, which is similar to Zong's report [39] where the transverse resonance peak shifts to shorter wavelength with the increase of the wire length. However, it is obviously different from Duan's report where the dipolar peak with a shorter wavelength was attributed to interband transition of Cu bulk metal, and the dipolar peak with a longer wavelength shifted to larger wavelengths with increasing wire length. In fact, the pores in the ion-track template are not aligned parallel but have a considerable angular distribution of 34° [50]; hence, some Cu nanowires filled in the template are not perpendicular to the template surface, as shown in Figure 2 in [42]. Therefore, some nanowires are not parallel to incident light though the incident light was perpendicular to the template surface. Based on these analyses, we suggest that the shorter dipolar peak should be the transverse dipole resonance of Cu NCs, and the longer dipolar peak should be the longitudinal resonance of Cu NCs, which displays a red shift with increasing the aspect ratio.

## Conclusions

Ag and Cu nanocrystals (NCs) were assembled into ordered porous anodic alumina (OPAA) by the single-potential-step chronoamperometry technique. For continuous electrodeposition, metallic nanowires are single crystalline with fcc structure; however, for interval electrodeposition, the nanowires are polycrystalline with bamboo-like or pearl-chain-like structure. The formation mechanisms of the nanoparticle nanowires and the single-crystalline nanowires were discussed in detail. The NCs/OPAA composite shows a significant SPR absorption. The transverse dipole resonance of metallic NCs enhances and has a blue shift with increasing electrodeposition time or the experimental cycle times. The NCs/OPAA composite will have promising applications in many fields related to the surface plasmon resonance of metal nanoparticles.

## Competing interests

The authors declare that they have no competing interests.

## Authors’ contributions

XY directed the research and finished the manuscript, JH carried out the synthesis and characteristics of Ag NCs/OPAA composite, YL carried out the synthesis and characteristics of Cu NCs/OPAA composite, and MC and WL participated in the studies. All authors read and approved the final manuscript.
